# Predictive model validation of new baseline renal function in southern Chinese patients after partial or radical nephrectomy

**DOI:** 10.7717/peerj.21306

**Published:** 2026-06-08

**Authors:** Jun Li, Gaoqiang Zhai, Yang Xie, Xian Yong Yan, Yan Qin, Bang Feng Liu, Shu Ting Tan, Qing Hua Gan, Wei Wang

**Affiliations:** 1 Department of Urology, Guangxi Hospital Division of The First Affiliated Hospital, Sun Yat-sen University, Nanning, China; 2 Department of Urology, The Second People’s Hospital of Nanning, Nanning, China; 3 Department of Urology, The People’s Hospital of Hechi, Nanning, China

**Keywords:** NB-GFR, Prediction, Radical nephrectomy, Partial nephrectomy

## Abstract

**Introduction:**

This study was designed to validate the formula of New Baseline Glomerular Filtration Rate (NB-GFR) within a multicenter cohort of southern Chinese patients who underwent radical nephrectomy (RN) or partial nephrectomy (PN), with a specific focus on assessing its accuracy and effectiveness.

**Methods:**

The predictive capability of the formula proposed by professor Palacios DA for estimating NB-GFR after PN or RN was evaluated, with a focus on its efficacy in the southern Chinese population. A retrospective analysis was conducted involving 398 RN patients and 185 PN patients from three hospitals between 2015 and 2022. The correlation between the postoperative NB-GFR predicted by the equation and the actual GFR of the patients was analyzed, and the level of agreement was assessed.

**Results:**

We found strong positive correlation between NB-GFR and GFR after PN, with a correlation coefficient of R = 0.645, 95% confidence interval (95% confidence interval (CI)) [0.552–0.722], *P* < 0.001. Moderate positive correlation was observed between NB-GFR and GFR after RN, with R = 0.434, 95% CI [0.339–0.501]. Lin’s concordance correlation coefficient (CCC) analysis demonstrated a moderate concordance between post-operative GFR and NB-GFR, with a CCC value of 0.522 (95% CI [0.461–0.579], *P* < 0.001) for RN and 0.533 (95% CI [0.472–0.589], *P* < 0.001) for PN.

**Conclusion:**

The NB-GFR prediction equation demonstrates moderate concordance with actual postoperative GFR in southern Chinese patients undergoing RN or PN, indicating broad application potential.

## Introduction

Renal cell carcinoma (RCC) is the most lethal malignancy in the urogenital system. The incidence of renal cancer in China was 4.99 per 100,000 in 2014, with 668,000 new cases and 234,000 deaths reported in 2015 (Siegel et al., 2012). Preoperative evaluation of renal function preservation following RCC surgery holds significant clinical importance (Pierorazio et al., 2016). Indeed, surgery-induced chronic kidney disease (CKD) patients have been shown to have long-term functional stability and favorable survival rates when the new New Baseline Glomerular Filtration Rate (NB-GFR) is ≥45 ml/min/1.73 m^2^ (Lane et al., 2015; Wu et al., 2018). When assessing the indications for partial nephrectomy (PN) *versus* radical nephrectomy (RN), the evaluation of NB-GFR is a crucial consideration. For small renal tumors, PN is the standard treatment because it better preserves renal function while achieving comparable oncological outcomes to RN. PN is also applicable to larger renal tumors if technically feasible (Ljungberg et al., 2019; Campbell et al., 2017). Maintaining long-term renal function stability can confer survival benefits, making the preservation of renal function a primary goal of PN. Consequently, renal function outcomes prediction following PN or RN was explored by numerous studies (Sorbellini et al., 2006; Kim et al., 2009; Liss et al., 2016; Shum et al., 2017; Martini et al., 2018; Bertolo et al., 2019; Bhindi et al., 2019; McIntosh et al., 2019; Aguilar Palacios et al., 2020). However, models proposed before are methodologically complex and have only moderate predictive capabilities. Certain models necessitate extra testing or resource compensation. Moreover, external validation is frequently absent, which makes the predicted results less suitable for clinical applications (Mir et al., 2015; Simmons et al., 2012; Tanaka et al., 2019; Funahashi et al., 2011).

In an effort to tackle this problem, Aguilar Palacios et al. (2020) devised and externally verified a simple algorithm. This algorithm is specifically crafted to precisely forecast the NB-GFR after either RN or PN. Notably, it can be easily applied in clinical settings, facilitating efficient preoperative patient counseling and providing appropriate guidance (Aguilar Palacios et al., 2021). The proposed equation model is exceptionally simple: the NB-GFR is predominantly influenced by the preoperative GFR, with limited contributions from tumor-related factors. Notably, the surgical procedure details, including whether the surgery is minimally invasive or open, or whether tumor ablation techniques were employed, are not incorporated into the equation. Nevertheless, the study cohort they utilized mainly consisted of non-Asian individuals. Specifically, in the largest cohort, 20% were African Americans and 77% were Caucasians. Moreover, as of now, the model has not undergone external validation outside of their initial study. This study evaluates the external validity of the NB-GFR prediction equation proposed by Aguilar Palacios et al. (2020) in a Southern Chinese cohort. The objective of this study was to validate the consistency between NB-GFR and actual post-operative eGFR, and simultaneously evaluate the predictive efficacy of the model for PN, RN, as well as the combined cohort without differentiating between PN and RN.

## Materials and Methods

This study aims to assess the predictive capacity of the equation proposed by Aguilar Palacios et al. (2020) for estimating postoperative NB-GFR in patients undergoing nephron-sparing surgery (NSS) or RN, with a particular focus on its applicability in the Southern Chinese population. To this end, we reviewed the general characteristics of patients included in the study and evaluated whether there were significant differences between the two groups. The study retrospectively analyzed patients who underwent RN or NSS from 2015 to 2022 in three hospitals in Guangxi. A multicenter cohort design was adopted, and all eligible cases during the concurrent period from three hospitals were enrolled to avoid single-center selective enrollment; meanwhile, baseline characteristics of the PN and RN subgroups were calculated to reduce intergroup confounding. A double data entry method was employed to verify the original data, and inconsistent entries were traced back to electronic medical records for reconfirmation; for subjective indicators, the interpretations were strictly based on the original descriptions in surgical records to avoid biases arising from manual judgment. During the initial enrollment phase of this study, the following cases were strictly excluded: ① patients with bilateral renal tumors; ② patients with preoperative solitary kidneys; ③ patients with hereditary tumor syndromes; ④ patients who underwent subsequent kidney-related surgeries after nephrectomy; ⑤ patients who were dialysis-dependent, or had insufficient medical records. Ultimately, 583 patients with non-metastatic renal tumors who underwent surgery were part of the study. Among them, 398 patients had RN and 185 patients had NSS. The core rationale for integrating data from three hospitals in this study is as follows: all three hospitals are grade-matched general hospitals located in southern China, and the regional, ethnic, and socioeconomic characteristics of the populations they serve are homogeneous, which ensures the representativeness of the cohort; in addition, all three institutions uniformly adopted surgical protocols formulated in accordance with the European Association of Urology guidelines for renal cell carcinoma and implemented consistent postoperative follow-up procedures. Clinical parameters for each patient were obtained retrospectively from electronic medical records and analyzed. In the present study, missing data primarily consisted of serum creatinine measurements obtained at 3–12 months postoperatively (with a missing rate of 6.2%) and partial clinical covariates (with a missing rate of 3.8%). For these missing data, multiple imputation was adopted for handling: an imputation model was constructed based on complete variables including age, sex, preoperative estimated glomerular filtration rate (eGFR), surgical approach, and tumor size. Subsequent statistical analyses pooled the results from each imputed dataset and reported the pooled effect sizes. Cases with missing data exceeding 20% (no such cases were observed in this study) were excluded directly. A total of 398 patients who underwent RN were included: the average age was 53.82 ± 12.29 years; males accounted for 68.60% (273 cases) and females for 31.40% (125 cases); the ethnic composition included 67.80% (270 cases) Han, 28.10% (112 cases) Zhuang, and 4.00% (16 cases) other minority groups; comorbid diabetes was present in 9.30% (37 cases), and non-diabetic patients accounted for 90.70% (361 cases); tumors larger than 7 cm were found in 29.40% (117 cases), with the remaining 70.60% (281 cases) having tumors smaller than 7 cm; average height was 165.00 (160.00–170.00)cm; average weight was 63.00 (55.00–72.00) Kg; average body mass index (BMI) was 23.44 (21.23–25.51) Kg/m^2^; preoperative serum creatinine was 83.00 (68.00–100.00) µmol/L; postoperative serum creatinine after 3–12 months was 109.00 (88.00–128.00) µmol/L; preoperative GFR averaged 42.33 (31.55–46.32) ml/min; postoperative GFR averaged 32.56 (28.91–39.85) ml/min; and average NB-GFR was 31.14 (25.09–36.89) ml/min. Among the 185 NSS patients: the average age was 50.65 ± 12.67 years; males accounted for 69.20% (128 cases) and females for 30.80% (57 cases); the ethnic composition included 68.60% (127 cases) Han, 28.10% (52 cases) Zhuang, and 3.20% (six cases) other minority groups; comorbid diabetes was present in 7.60% (14 cases), and non-diabetic patients accounted for 92.40% (171 cases); tumors larger than 7 cm were found in 2.70% (five cases), with the remaining 97.30% (180 cases) having tumors smaller than 7 cm; average height was 165.00 (159.25–170.00)cm; average weight was 64.00 (56.00–73.00) Kg; average BMI was 23.89 (21.49–26.16) Kg/m^2^; preoperative serum creatinine was 78.00 (66.00–94.00) µmol/L; postoperative serum creatinine after 3–12 months was 87.00 (71.50–106.50) µmol/L. For the detection of serum creatinine, fasting morning venous blood samples were collected from all patients, and the measurement time points were strictly consistent with the scheduled postoperative follow-up time window (3–12 months), which effectively eliminated the interference of confounding factors such as diet and physical activity on the detection results. In terms of multicenter laboratory quality control, the three participating institutions conducted quarterly external quality assessment (EQA) for serum creatinine detection, and the EQA qualification rate reached 100% during the study period of 2015–2021. Preoperative GFR averaged 46.71 (38.55–53.89) ml/min; postoperative GFR averaged 43.78 (35.21–48.59) ml/min; and average NB-GFR was 52.45 (45.64–58.1) ml/min. The surgeries were performed by experienced urologists, with the surgical approach (transperitoneal or retroperitoneal) was determined based on the tumor location. Under warm ischemia, tumor resection was carried out. The approach of NSS, including partial nephrectomy or tumor enucleation, was selected according to the surgeon’s discretion. For RN, the surgery was performed either *via* a minimally invasive technique or an open surgical approach, which was determined by the tumor’s features and the patient’s overall condition. Written informed consent was secured from all participating patients. Ethics Committee of Guangxi Hospital Division of The First Affiliated Hospital, Sun Yat-sen University approved this project, the approval number is KY-KJT-2023-031.

NB-GFR was defined as the last available estimated glomerular filtration rate (eGFR) measurement obtained within the 3–12-month postoperative window. This time window was selected because renal function typically stabilizes at 3 months after surgery, and measurements within 3–12 months can reliably reflect the stable new baseline renal function, thus avoiding the interference of early postoperative renal function fluctuations. Estimation of eGFR: for all patients, preoperative and postoperative serum creatinine levels were collected, and the eGFR (including preoperative eGFR and actual postoperative eGFR) was calculated using the Chronic Kidney Disease Epidemiology Collaboration (CKD-EPI) equation, with variables including age, gender, ethnicity and serum creatinine, which are routinely available in clinical practice. Prediction of NB-GFR: The predicted NB-GFR was calculated using the dedicated formula proposed by Aguilar Palacios et al. (2020), and the formula is as follows: 35 + preoperative eGFR × 0.65 − 18 (for radical nephrectomy [RN]) − Age × 0.25 + 3 (if tumor size > 7 cm) − 2 (if the patient has comorbid diabetes). The general characteristics of the 398 patients undergoing RN and the 185 patients undergoing PN were summarized and statistically analyzed to determine significant differences between the two groups. The evaluation indicators included age, gender, ethnicity, diabetes status, tumor size (>7 cm or not), height, weight, BMI, preoperative and postoperative serum creatinine levels, preoperative and postoperative GFR, and NB-GFR for both groups. The NB-GFR for patients undergoing RN or PN was calculated using the proposed equation.

For continuous data, if the data meet the normal distribution, the independent samples *t*-test is used for comparisons between two groups, and one-way analysis of variance (ANOVA) is used for comparisons involving more than two groups. In cases where the data fail to conform to a normal distribution, they are presented in the format of median (minimum, maximum), denoted as (M(min, max)). For comparing data between two groups under such circumstances, the Mann–Whitney U test is employed. When the comparison involves more than two groups, the Kruskal–Wallis H test is utilized. For categorical data, the chi-square test is employed.The correlation between NB-GFR and the actual postoperative GFR measured in patients was analyzed using both Pearson’s correlation coefficient and Spearman’s rank correlation coefficient to evaluate the relationship between the NB-GFR derived from the aforementioned equation and the actual postoperative GFR. Lin’s concordance correlation coefficient (CCC) and Bland-Altman analysis were further used to evaluate the consistency between the postoperative GFR and the NB-GFR in patients undergoing PN or RN, to further validate the accuracy of the NB-GFR. Calibration analyses were conducted in the RN and PN groups to validate the accuracy of model. All statistical analyses were performed using SPSS version 22.0 and R version 4.1.3.

## Results

There were no statistically significant differences in gender, ethnicity, diabetes status, height, weight, or BMI between the RN and PN groups. However, statistically significant differences were observed in age, tumor size, preoperative serum creatinine, 3–12 month postoperative serum creatinine, preoperative GFR, postoperative GFR, and NB-GFR (all *P* < 0.05) (Table 1). Laparoscopic surgery was performed in 393 patients, robot-assisted laparoscopic surgery in 125 patients, and open surgery in 65 patients. Among the 583 enrolled patients, 65 were diagnosed with CKD: 30 at CKD stage 1, 25 at stage 2, and 10 at stage 3. These between-group differences were related to baseline and surgical characteristics but did not materially affect the concordance between NB-GFR and actual postoperative GFR.

**Table 1:** Baseline information of patients receiving RN and PN.

	RN (*n* = 398)	PN (*n* = 185)	t/u/*χ*2	*P*
Age	53.82 ± 12.29	50.65 ± 12.67	2.875	0.004
Gender			0.021	0.885
Male	273 (68.60%)	128 (69.20%)		
Female	125 (31.40%)	57 (30.80%)		
Ethnicity			0.214	0.898
Han	270 (67.80%)	127 (68.60%)		
Zhuang	112 (28.10%)	52 (28.10%)		
Others	16 (4.00%)	6 (3.20%)		
Diabetes			0.473	0.492
Yes	37 (9.30%)	14 (7.60%)		
No	361 (90.70%)	171 (92.40%)		
Tumor size>7 cm			54.387	<0.001
Yes	117 (29.40%)	5 (2.70%)		
No	281 (70.60%)	180 (97.30%)		
Renal score	8.95 ± 1.68	6.72 ± 1.25	18.76	<0.001
Height (cm)	165.00 (160.00∼170.00)	165.00 (159.25∼170.00)	−0.181	0.856
Weight (Kg)	63.00 (55.00∼72.00)	64.00 (56.00∼73.00)	−1.14	0.254
BMI	23.44 (21.23∼25.51)	23.89 (21.49∼26.16)	−1.482	0.138
Preoperative serum creatinine (umol/L)	83.00 (68.00∼100.00)	78.00 (66.00∼94.00)	−2.179	0.029
Serum creatinine at 3–12 months postoperatively (umol/L)	109.00 (88.00∼128.00)	87.00 (71.50∼106.50)	−7.233	<0.001
Preoperative GFR	42.33 (31.55∼46.32)	46.71 (38.55∼53.89)	−2.336	0.036
Post-operative GFR	32.56 (28.91∼39.85)	43.78 (35.21∼48.59)	−6.733	<0.001
NB-GFR	31.14 (25.09∼36.89)	52.45 (45.64∼58.1)	−16.49	<0.001

**Figure 1:**
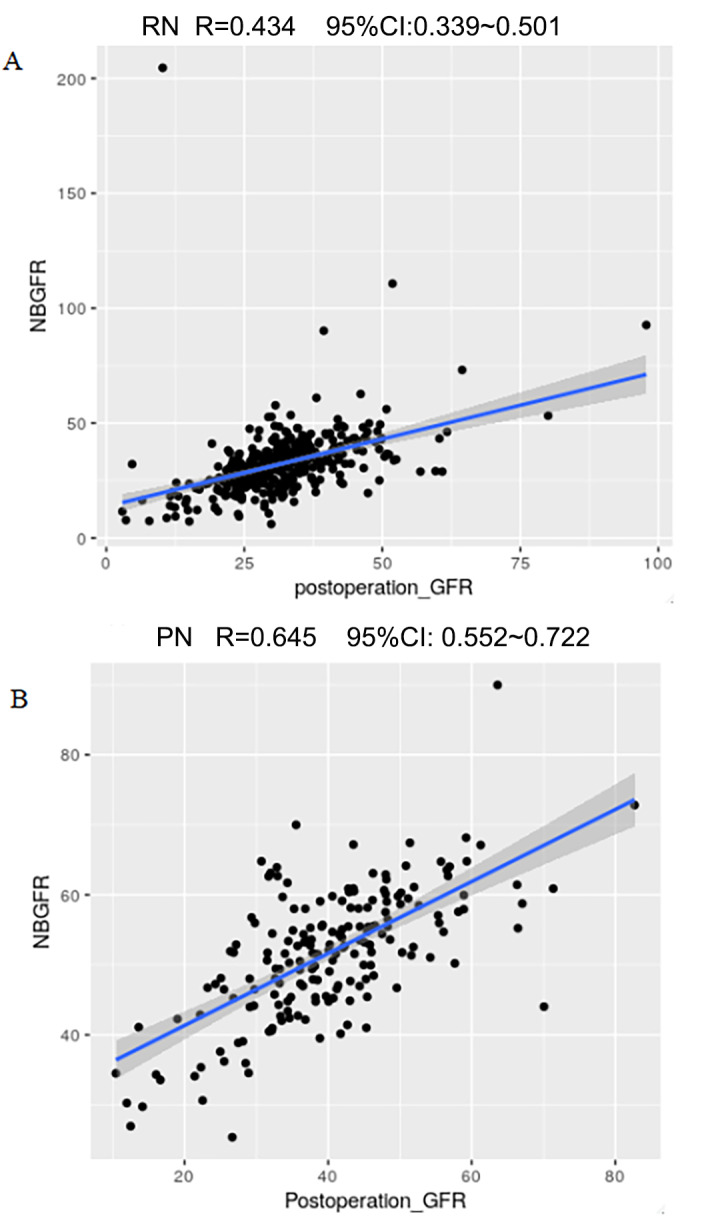
Pearson’s correlation analysis for postoperative GFR and NB-GFR. (A) For patients undergoing RN, a moderate positive correlation was found between postoperative GFR and NB-GFR, with a correlation coefficient of 0.434, and a 95% CI [0.339–0.501]; (B) For patients undergoing PN, a strong positive correlation was found between postoperative GFR and NB-GFR, with a correlation coefficient of 0.645, and a 95% CI [0.552–0.722].

Upon statistical analysis, a linear relationship was observed between NB-GFR and postoperative GFR. To assess the correlation between the NB-GFR derived from the above equation and the actual postoperative GFR, both Pearson’s correlation analysis and Spearman’s rank correlation analysis were employed. Firstly, Pearson’s correlation was used to evaluate the relationship between postoperative GFR and NB-GFR. For patients undergoing PN, a strong positive correlation was found between postoperative GFR and NB-GFR, with a correlation coefficient of 0.645, and a 95% confidence interval (CI) [0.552–0.722], with *P* < 0.001 (Fig. 1A). In contrast, for patients undergoing RN, the correlation was weaker, with an R value of 0.434 and a 95% CI [0.339–0.501], also with *P* < 0.001 (Fig. 1B). Next, Spearman’s rank correlation was applied to evaluate the relationship between postoperative GFR and NB-GFR. A moderate positive correlation was observed between postoperative GFR and NB-GFR for RN patients, with an R value of 0.512 and a 95% CI [0.436–0.581], with *P* < 0.001. Similarly, for PN patients, a moderate positive correlation was found, with an R value of 0.602 and a 95% CI [0.501–0.688], also with *P* < 0.001.

**Figure 2:**
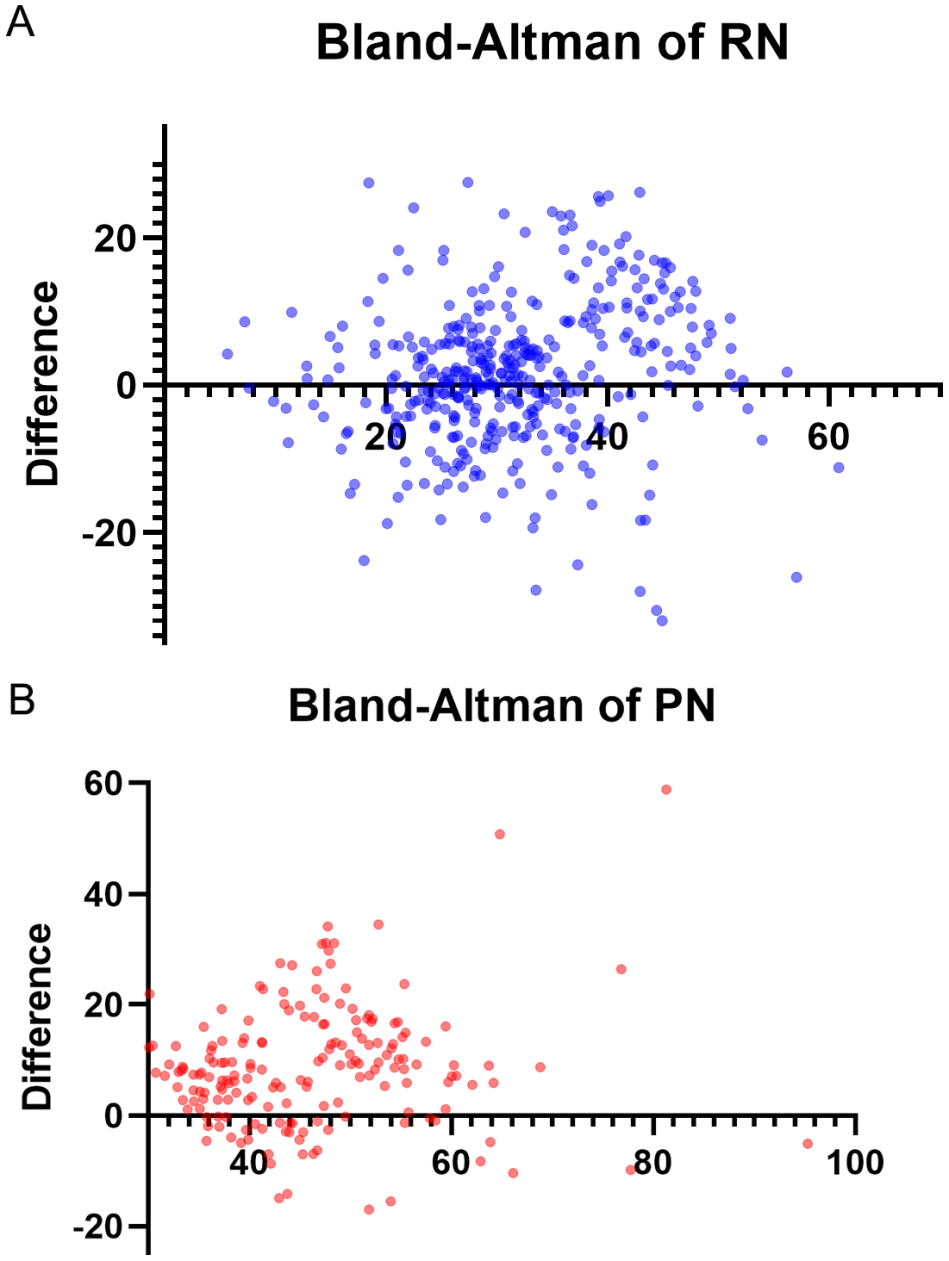
Bland-Altman analysis for postoperative GFR and NB-GFR. (A) The bias between postoperative GFR and NB-GFR in RN was 1.622, standar difference of bias was 9.777, 95% limits of agreement was −17.54∼20.78; (B) The bias between postoperative GFR and NB-GFR in PN was 9.577, standar difference of bias was 17.67, 95% limits of agreement was −25.05∼44.21.

**Figure 3:**
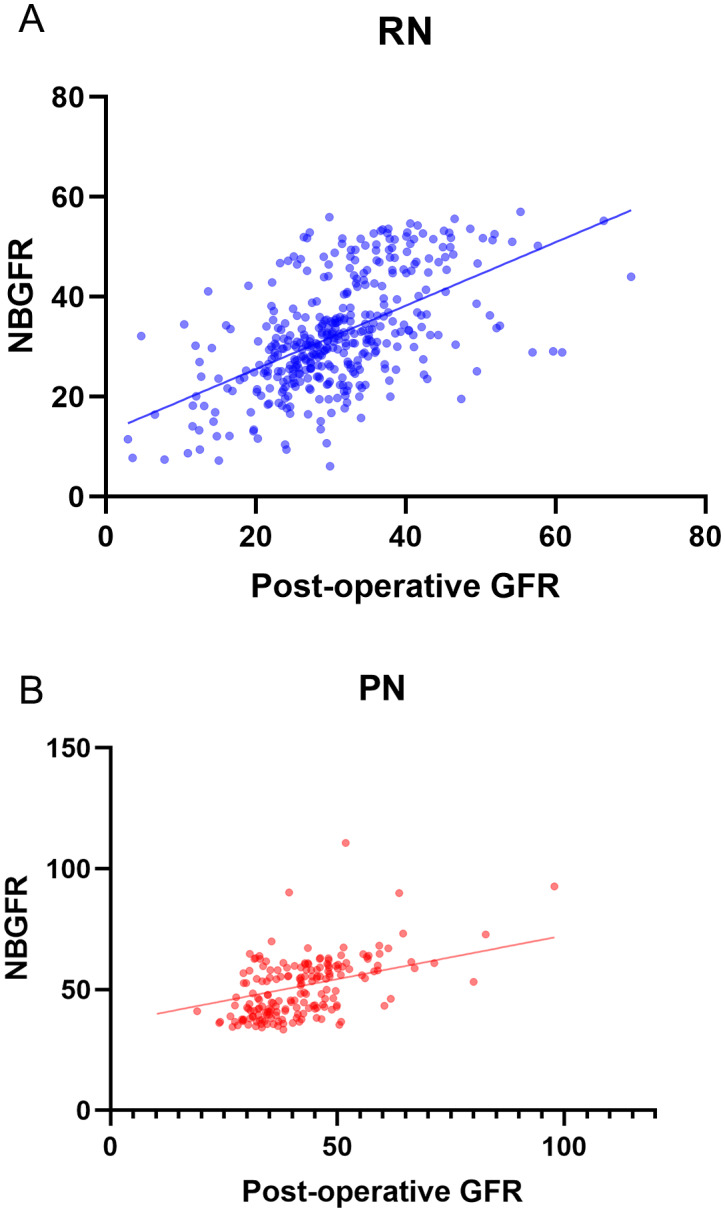
Calibration analysis for postoperative GFR and NB-GFR. (A) Calibration slope in RN group between NB-GFR and post-operative GFR was 0.7232, intercept was 12.84; (B) Calibration slope in PN group between NB-GFR and post-operative GFR was 0.5610, intercept was 18.76.

After performing correlation analyses using different methods, it was evident that there is a strong correlation between postoperative GFR and NB-GFR, regardless of whether the patients underwent RN or PN. CCC analysis revealed that the CCC value for patients who underwent RN between postoperative GFR and NB-GFR was 0.522, 95% CI [0.461∼0.579], with *P* < 0.001. Regarding PN group, the CCC value was 0.533, 95% CI [0.472–0.589], with *P* < 0.001. As for Bland-Altman analysis for RN, the bias between postoperative GFR and NB-GFR was 1.622, standard deviation of the differences was 9.777, 95% limits of agreement were −17.54 to 20.78 (Fig. 2A). For PN group, the bias between postoperative GFR and NB-GFR was 9.577, standard deviation of the differences was 17.67, 95% limits of agreement were −25.05–44.21 (Fig. 2B). Calibration analyses showed that calibration slope in RN group between NB-GFR and post-operative GFR was 0.7232, intercept was 12.84 (Fig. 3A). In PN group, calibration slope between NB-GFR and post-operative GFR was 0.5610, intercept was 18.76 (Fig. 3B).

## Discussion

In the past few years, numerous studies have put forward predictive models for postoperative renal function. Nevertheless, these pre-existing models frequently incorporate a multitude of factors and are excessively intricate, rendering them unfit for clinical application (Sorbellini et al., 2006; Liss et al., 2016; Shum et al., 2017; Martini et al., 2018; Bhindi et al., 2019; Aguilar Palacios et al., 2020). Aguilar Palacios et al. (2021) proposed the following formula to predict postoperative GFR: 35 + preoperative GFR (×0.65)−18 (if RN)−age (×0.25) + 3 (if tumor size > 7 cm) −2 (if diabetes is present). This equation-based model is comparatively straightforward and pragmatic. It only includes five preoperative factors, namely age, the existence of diabetes, preoperative glomerular filtration rate (GFR), tumor size, and the type of surgery (PN or RN). The objective of this research is to verify the predictive capacity of this simplified equation model for the NB-GFR following PN or RN. Additionally, we aim to conduct external validation of the model using data collected from a population in southern China. The core objective of this study is to assess the accuracy of the equation when applied to the population in southern China.

A total of 398 patients who underwent RN and 185 patients who underwent PN were included in the study. The baseline characteristics comparison showed no statistically significant differences in gender, ethnicity, diabetes status, height, weight and BMI between the two groups, while significant differences were observed in age, tumor size, preoperative and postoperative serum creatinine, preoperative and postoperative GFR as well as NB-GFR (all *P* < 0.05). Preoperative data were collected according to the content of the equation, and NB-GFR was calculated. Correlation analysis was performed between the calculated NB-GFR and the actual postoperative GFR. The results showed that there was a strong correlation between postoperative GFR and NB-GFR for both RN and PN patients. Furthermore, the equation was found to be more accurate in predicting NB-GFR after PN compared to RN. CCC analysis further verified the linear correlation between NB-GFR calculated by the formula and actual postoperative GFR. In the RN group, the CCC was 0.522 (*P* < 0.001), and in the PN group, the CCC was 0.533 (*P* < 0.005). In conclusion, the results of this study indicate that the equation model provides a moderate level of consistency between the predicted NB-GFR and the actual postoperative GFR.

The initial study population for the equation model primarily consisted of Caucasians (77%) and African Americans (20%). Using serum creatinine to estimate GFR in African Americans might introduce errors due to racial differences, and the proportion of Asian patients included in the original study was small (<3%). Racial disparities might have an impact on the predictive power of the equation. Consequently, we applied this equation to an Asian cohort, specifically in southern China. To accommodate regional and racial discrepancies, we employed the CKD-EPI to estimate the GFR, with the goal of enhancing the accuracy of the equation within the Chinese population. Intriguingly, in contrast to other predictive models, this equation does not incorporate variables like surgical particulars. The validation of the equation by Hidekazu and colleagues in a Japanese population who had RN or PN showed that tumor size (<2 cm, 2–4 cm, or >4 cm) and complexity (low, moderate, or high) did not alter its accuracy (Tachibana et al., 2022). Techniques in surgery that aim to reduce parenchymal volume loss or ischemic time might have modest benefits for the kidney’s long-term function. Additionally, factors such as age, preoperative GFR, and diabetes, which are related to the patient, are likely the primary determinants of kidney function after surgery. However, validation studies of this equation in the Chinese population, particularly in southern China, have not been reported. The merit of this equation is its preoperative predictive ability for the NB-GFR. This capability not only aids in more effectively communicating with patients the risk of developing CKD or needing dialysis after the operation but, more crucially, enables a more accurate evaluation of the advantages of RN compared to PN. This is especially relevant in scenarios where performing PN for renal tumors might affect oncological outcomes, or when RN could lead to a postoperative GFR of less than 45 ml/min/1.73 m^2^.

## Conclusion

In patients from southern China who undergo RN or PN, the NB-GFR equation shows a significant positive correlation with measured GFR in terms of changing trends and exhibits a moderate level of numerical agreement. Its predictive efficacy is superior in patients undergoing nephron-sparing PN compared to those undergoing RN, and it can serve as a reference tool for the preliminary assessment of postoperative renal function in this population.

## Limitation

The relatively high correlation coefficient observed in this study only reflects the consistency in the changing trends of predicted and measured GFR between predicted GFR and measured GFR, and does not indicate predictive precision. Besides, this study has inherent limitations due to its retrospective design, which cannot fully eliminate residual confounding factors such as the impact of postoperative medications on renal function and may introduce unmeasured biases into GFR-related analyses. The initial analysis lacked calibration metrics, a critical oversight that could lead to misjudgment of the NB-GFR equation’s overall efficacy given that calibration is essential for the reliable clinical application of predictive models. Although the NB-GFR equation (originally developed for Western populations) was validated for southern Chinese populations in this study, its generalizability to northern Chinese populations remains unconfirmed and requires further verification. The fundamental biological differences between RN (irreversible nephron loss) and PN (renal parenchyma preservation), which result in distinct renal function recovery patterns, explain the lower accuracy of the NB-GFR equation in the RN subgroup and necessitate further validation stratified by surgical modality in future research.

##  Supplemental Information

10.7717/peerj.21306/supp-1 Supplemental Information 1 Raw data

## Additional Information and Declarations

### Data Availability

The following information was supplied regarding data availability: Raw data is available in the Supplemental File.
